# Atrial leadless pacing for heart failure in sick sinus syndrome

**DOI:** 10.1016/j.jccase.2026.07.007

**Published:** 2026-08-08

**Authors:** Shinnosuke Sato, Yasuyuki Takada, Junichi Kamoshida, Muryo Terasawa, Kazuhiro Satomi, Yoshinao Yazaki

**Affiliations:** Department of Cardiology, Tokyo Medical University, Tokyo, Japan

**Keywords:** Leadless pacemaker, Sick sinus syndrome, Heart failure, Subcutaneous implantable cardioverter-defibrillator, Atrioventricular synchrony

## Abstract

Two patients with sick sinus syndrome and heart failure were treated with atrial leadless pacing. Case 1 demonstrated hemodynamic improvement with cardiac output increase (3.0 → 5.1 L/min), B-type natriuretic peptide (BNP) reduction (3223 → 480 pg/mL), and New York Heart Association class improvement (III → I) following Aveir dual-chamber leadless pacemaker (Abbott Medical, Inc., Plymouth, MN, USA) implantation. Case 2, at high infection risk with mechanical valve and prior endocarditis, received hybrid therapy combining atrial fibrillation ablation, Aveir atrial pacing, and subcutaneous implantable cardioverter-defibrillator implantation, achieving left ventricular ejection fraction improvement (20 → 38%) and BNP reduction (321 → 28 pg/mL). Atrial leadless pacing may represent a therapeutic option in selected patients with preserved atrioventricular conduction or high infection risk requiring physiologic pacing.

**Learning objective:**

This case report demonstrates that atrial leadless pacing can be an effective treatment option for patients with sick sinus syndrome complicated by heart failure when conventional transvenous atrial pacing is undesirable. In one of the presented cases, the feasibility of combining a leadless pacemaker with a subcutaneous implantable cardioverter-defibrillator is also illustrated, providing insight into a potential lead-free device strategy.

## Introduction

Leadless pacemaker technology initially provided only single-chamber ventricular pacing. Ventricular pacing can induce dyssynchrony and exacerbate heart failure, resulting in increased hospitalizations compared to atrial-based strategies [1]. The development of atrial leadless pacemakers may address this limitation. We report two cases demonstrating atrial leadless pacing efficacy for heart failure management in sick sinus syndrome, emphasizing patient selection and device coordination strategies.

## Case report

### Case 1: hemodynamic-guided atrial pacing strategy

An 83-year-old woman with a history of hypertension, hypertrophic cardiomyopathy, moderate-to-severe aortic regurgitation, and sick sinus syndrome presented with recurrent heart failure hospitalizations despite optimal medical therapy. Invasive hemodynamic assessment during cardiac catheterization demonstrated that cardiac output increased from 3.0 L/min (junctional rhythm, 40 bpm) to 5.1 L/min (atrial pacing, 70 bpm), with pulmonary capillary wedge pressure reduction from 21 to 12 mmHg (Fig. 1).

**Fig. 1:**
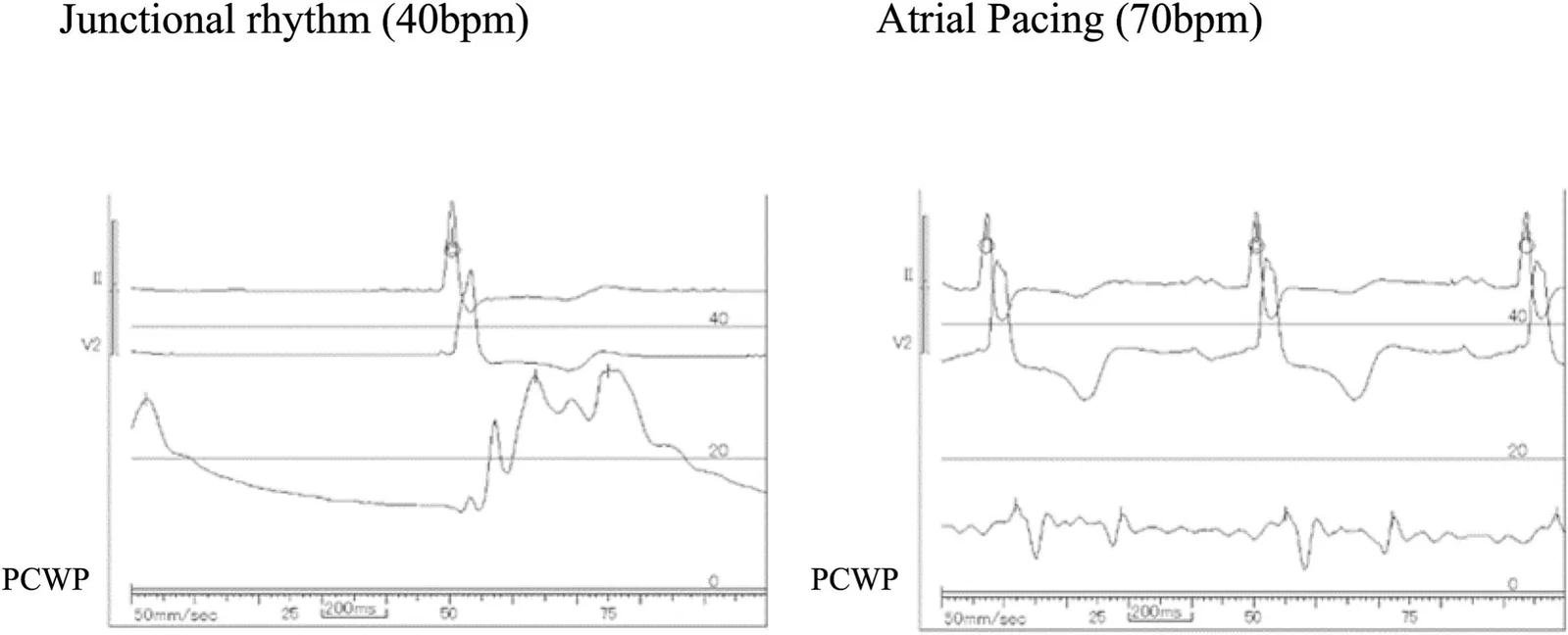
Invasive hemodynamic assessment during cardiac catheterization in Case 1. Representative pulmonary capillary wedge pressure (PCWP) waveforms obtained during junctional rhythm at 40 bpm and atrial pacing at 70 bpm are shown. Atrial pacing increased cardiac output from 3.0 to 5.1 L/min and reduced pulmonary capillary wedge pressure from 21 to 12 mmHg.

Based on these findings, Aveir dual-chamber leadless pacemaker (Abbott Medical, Inc., Plymouth, MN, USA) was implanted via a femoral vein approach and programmed to AAIR+VVI mode 60/40 bpm. Although atrial pacing alone appeared hemodynamically effective, a dual-chamber leadless system was selected to provide back-up ventricular pacing in case of future atrioventricular conduction deterioration and to maintain physiologic pacing flexibility. The permanent lower rate was set at 60 bpm to avoid excessive resting tachycardia in this elderly patient. During 5-month follow-up, New York Heart Association (NYHA) functional class improved from III to I and serum B-type natriuretic peptide (BNP) progressively declined from 3223 to 480 pg/mL, with freedom from rehospitalization (Fig. 2).

**Fig. 2:**
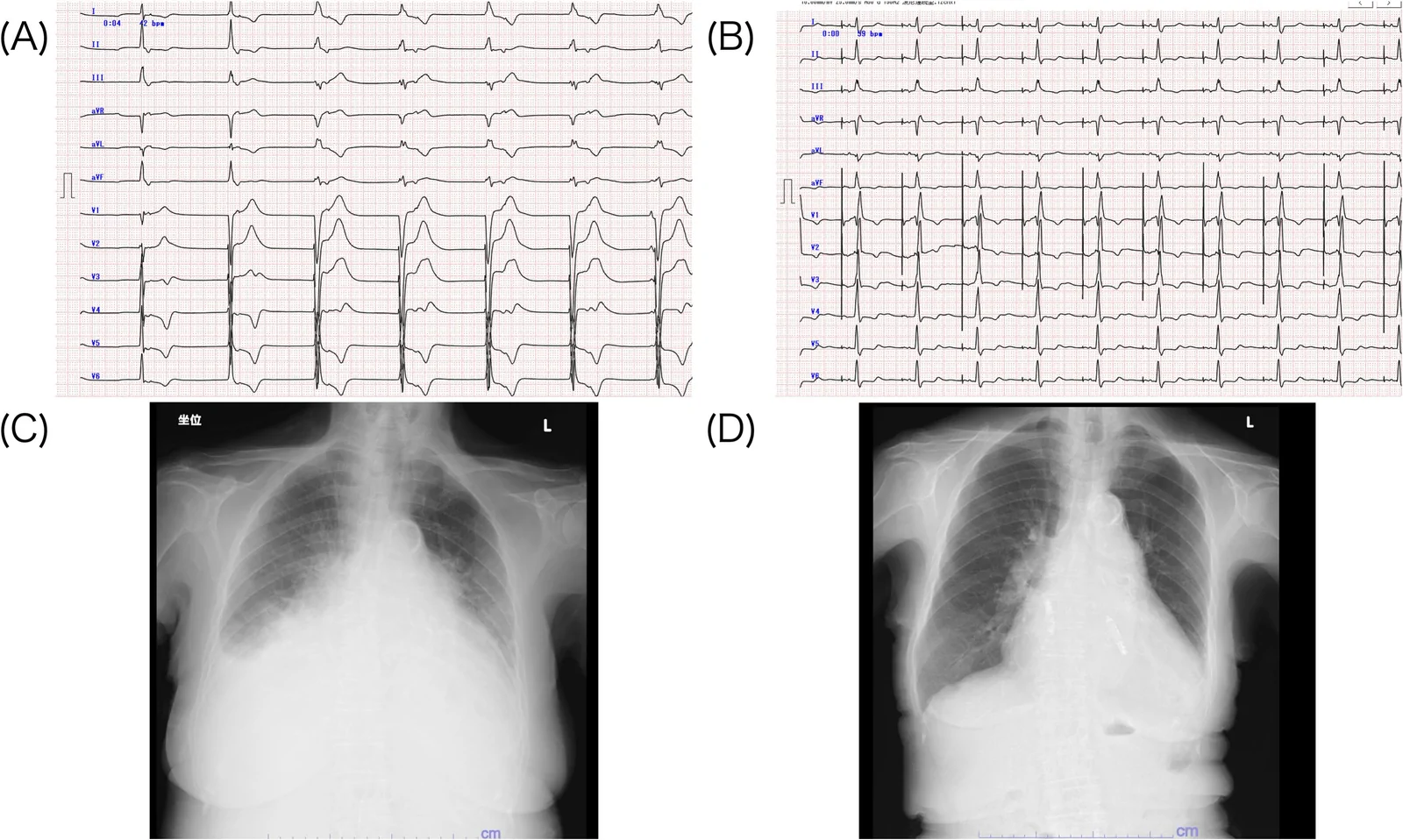
Electrocardiograms (A: before implantation, B: after implantation) and chest radiographs (C: before implantation, D: after implantation) showing atrial leadless pacemaker implantation in Case 1, demonstrating successful atrial pacing with improved heart rate and preserved intrinsic atrioventricular conduction.

### Case 2: hybrid leadless system in high-risk patient

A 69-year-old man with a history of mechanical mitral valve, prior endocarditis requiring valve re-replacement, and dilated cardiomyopathy [left ventricular ejection fraction (LVEF) 20%] developed atrial fibrillation precipitating heart failure exacerbation and out-of-hospital cardiac arrest due to ventricular fibrillation. Because of the patient's prior infective endocarditis, prosthetic valve, and the anticipated high risk of device-related infection, a transvenous lead-free strategy using subcutaneous implantable cardioverter-defibrillator (S-ICD) (Boston Scientific, Marlborough, MA, USA) and atrial leadless pacing was preferred over conventional transvenous ICD implantation. Therefore, a hybrid approach was employed: simultaneous atrial fibrillation catheter ablation with Aveir atrial leadless pacing, followed by S-ICD implantation.

Defibrillation threshold (DFT) testing confirmed appropriate S-ICD function without leadless pacemaker interference. Pre-implant electrocardiogram showed junctional rhythm (QRS 160 ms), while post-implant atrial pacing resulted in narrower QRS duration (110 ms), demonstrating preserved intrinsic atrioventricular conduction. Pre-implant S-ICD screening was performed according to standard protocol, and no inappropriate sensing was observed during implantation or short-term follow-up. At 3-month follow-up, atrial pacing (60 bpm) resulted in BNP reduction from 321 to 28 pg/mL and LVEF improvement from 20% to 38%, enabling beta-blocker optimization (Fig. 3).

**Fig. 3:**
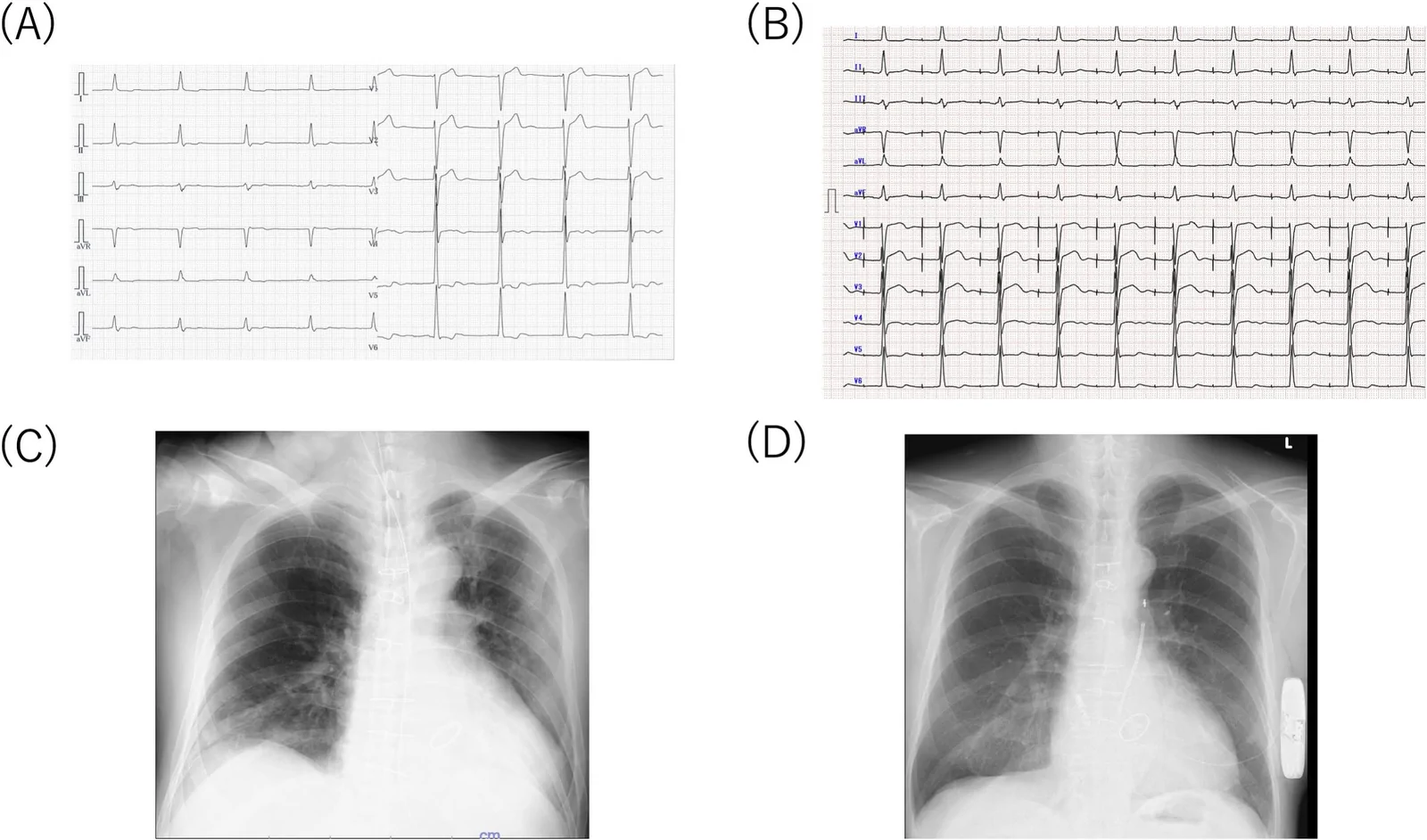
Electrocardiograms (A: before implantation, B: after implantation) and chest radiographs (C: before treatment, D: during follow-up) in Case 2. Pre-implant electrocardiogram demonstrated junctional rhythm, whereas post-implant electrocardiogram showed atrial pacing with preserved intrinsic atrioventricular conduction and narrower QRS duration. Chest radiographs before treatment (C) and during follow-up (D) demonstrated improvement in cardiomegaly and pleural effusion.

## Discussion

Recent rapid development of cardiac implantable electronic devices has provided several therapeutic options for individualized management of both brady- and tachyarrhythmias. Leadless pacemakers are particularly advantageous in patients at risk of device-related infection during long-term use of transvenous lead systems. In patients with sick sinus syndrome and heart failure, atrial pacing without atrioventricular dyssynchrony may provide favorable hemodynamic effects. Before the Aveir DR system, leadless pacing had been limited to ventricular pacing or atrial-sensing ventricular pacing systems [2]. The recent development of atrial leadless pacing systems has expanded treatment options for patients requiring physiologic pacing while avoiding complications related to transvenous leads.

In the first case, invasive hemodynamic assessment during cardiac catheterization provided direct, patient-specific evidence that atrial pacing was hemodynamically beneficial prior to device implantation. The observed improvement—increased cardiac output from 3.0 to 5.1 L/min and reduced pulmonary capillary wedge pressure from 21 to 12 mmHg during atrial pacing at 70 bpm—supported the indication for permanent atrial pacing. While heart rate augmentation contributed to the improvement in cardiac output, the concomitant reduction in pulmonary capillary wedge pressure suggests an additional hemodynamic contribution from restored atrioventricular synchrony and atrial contraction, consistent with prior hemodynamic studies [3], [4]. This approach of confirming hemodynamic benefit invasively before implantation may help guide patient selection in complex cases where the clinical benefit of atrial pacing is uncertain.

Alternative pacing strategies including dual-chamber pacing, conduction system pacing (CSP), and cardiac resynchronization therapy (CRT) warrant consideration in patients with sick sinus syndrome complicated by heart failure, and the rationale for device selection requires case-specific justification. In Case 1, the patient had preserved atrioventricular conduction with hypertrophic cardiomyopathy and moderate-to-severe aortic regurgitation. Given the risk of dyssynchrony-related pacing-induced cardiomyopathy with right ventricular pacing, atrial-only pacing with back-up ventricular pacing was preferred. CSP was not deemed necessary given intact atrioventricular conduction, and CRT was not indicated in the absence of ventricular dyssynchrony with preserved ejection fraction. In Case 2, the prohibitively high infection risk imposed by a mechanical prosthetic valve and prior infective endocarditis made any transvenous lead strategy unacceptable. The atrial leadless pacemaker was required in addition to S-ICD to restore atrioventricular synchrony after atrial fibrillation ablation and to enable beta-blocker optimization, which subsequently contributed to LVEF recovery.

There are several limitations of current leadless pacing systems in managing patients with tachyarrhythmia. The Aveir pacing system does not currently provide arrhythmia storage, anti-tachycardia pacing, or remote monitoring. Conduction system pacing is also unavailable, limiting its use in patients requiring ventricular pacing with dyssynchrony or those at risk for pacing-induced cardiomyopathy. In addition, long-term clinical data regarding atrial leadless pacing remain limited.

The combination of S-ICD and leadless pacemaker without transvenous leads represents an attractive approach for high-risk populations, including those with prosthetic valves, prior device infections, or compromised venous access. In the second case, the hybrid approach using S-ICD and Aveir AR was successfully applied in a patient with bradycardia-induced heart failure and life-threatening ventricular arrhythmia. Stable atrial pacing enabled optimization of medical therapy and improvement in cardiac function during follow-up.

Potential device interactions between S-ICD and Aveir systems have been a concern in simultaneous use. To the best of our knowledge, only a few cases of combined S-ICD and Aveir use have been previously reported [5], [6], [7]. In our experience, device interaction was evaluated not only intra-procedurally during defibrillation threshold testing but also during outpatient follow-up using electrocardiographic assessment and device interrogation. No inappropriate sensing was observed during short-term follow-up. However, the potential risk of T-wave oversensing, particularly during recurrent atrial fibrillation, cannot be completely excluded. Nevertheless, long-term interaction between S-ICD and atrial leadless pacing systems remains insufficiently characterized. With the upcoming EMPOWER leadless pacemaker (Boston Scientific), further combinations of leadless pacemakers and S-ICD are expected, and device compatibility may become more reliable given that both systems are developed by the same company.

This report has several limitations. First, only two cases were included with relatively short follow-up duration, and the generalizability of these findings remains uncertain. Second, long-term interaction between the S-ICD and leadless pacing systems could not be fully evaluated, and the risk of T-wave oversensing during future atrial fibrillation recurrence cannot be excluded. Third, in Case 1, the observed hemodynamic and clinical improvement may partially reflect heart rate optimization itself rather than a device-specific effect of the atrial leadless pacemaker. The contribution of restored atrioventricular synchrony beyond rate increase could not be formally isolated in this case. Fourth, in Case 1, the hemodynamic assessment was performed at 70 bpm while the permanent rate was set at 60 bpm, and direct hemodynamic validation at the programmed rate was not obtained. These limitations should be considered when interpreting the clinical implications of our findings.

These cases demonstrate that atrial leadless pacing can be a reasonable therapeutic strategy for heart failure management in carefully selected patients with sick sinus syndrome, particularly when avoidance of transvenous leads is clinically desirable. The use of invasive hemodynamic assessment to guide implantation decisions may serve as a practical model for patient selection in ambiguous cases. Further prospective studies are needed to clarify the optimal patient profiles for atrial leadless pacing, its long-term outcomes, and the safety of hybrid leadless systems combining atrial pacing with S-ICD therapy.

## Consent statement

Written informed consent was obtained from both patients for participation and publication, including clinical data and images.

## Declaration of Generative AI and AI-assisted technologies in the writing process

During the preparation of this work, the authors used ChatGPT (OpenAI) solely for English language editing and grammar checking. After using this tool, the authors reviewed and edited the content as necessary and take full responsibility for the content of the published article.

## Declaration of competing interest

The authors declare that there is no conflict of interest.
